# Comparison on efficacy of radical prostatectomy versus external beam radiotherapy for the treatment of localized prostate cancer

**DOI:** 10.18632/oncotarget.20078

**Published:** 2017-08-09

**Authors:** Linyan Chen, Qingfang Li, Yexiao Wang, Yiwen Zhang, Xuelei Ma

**Affiliations:** ^1^ State Key Laboratory of Biotherapy and Cancer Center, West China Hospital, Sichuan University and Collaborative Innovation Center, Chengdu, PR China

**Keywords:** prostate cancer, external beam radiotherapy, surgery, meta-analysis

## Abstract

Since there was no consensus on treatment options of localized prostate cancer, a meta-analysis was performed to compare the efficacy of radical prostatectomy (RP) versus external beam radiotherapy (EBRT) concluding three-dimensional conformal radiotherapy (3DCRT) and intensity-modulated radiation therapy (IMRT). The search of eligible studies was performed on PubMed and Embase databases. The overall survival (OS), cancer-specific survival (CSS) and biochemical disease-free survival (BDFS) were compared by hazard ratio (HR) and odd ratios (OR). Twelve studies with 17137 patients were included. The pooled HR and 95% CI for OS, CSS and BDFS were 1.60 (1.44–1.79), 1.73 (1.34–2.24) and 0.65 (0.51–0.82), respectively. However, according to risk stratification, the HRs of CSS for low- to intermediate-risk patients were not significant. The 5-year and 10-year CSS reported significant OR and 95% CI of 1.96 (1.42–2.72) and 2.44 (1.33–4.48), except for 2-year CSS (*P =* 0.42). In conclusion, RP was generally associated with decreased risk of overall and cancer-specific mortality as well as better 5-year and 10-year OS and CSS. The EBRT was suggested to be a promising alternative option for low- to intermediate-risk patients. Large-scale prospective studies with risk stratification and adequate follow-up length were needed for further comprehensive comparison.

## INTRODUCTION

As the second most common cancer, prostate cancers are the sixth leading cause of cancer death in males with 1,112,000 confirmed cases and 307,000 deaths worldwide in 2012 [1, 2]. Radical prostatectomy (RP) and radiotherapy have been considered as recommended treatments to decrease the rate of cancer mortality and progression for patients with localized prostate cancer [3, 4]. Nevertheless, which treatment is more effective remains an open question.

For the past two decades, the external beam radiotherapy (EBRT) has developed rapidly from two-dimensional planning with X-rays films to three-dimensional conformal RT (3DCRT), intensity-modulated radiation therapy (IMRT) and etc. [5]. The 3DCRT was capable to deliver a conformal radiation dose without raised exposure of surrounding healthy areas [6]. Compared with 3DCRT, the IMRT achieved more conformal irradiation to target area with increased and uniform distributed radiation dose, while minimizing radiation-induce side effects [7]. In addition, with the development of laparoscopy and robotic surgery techniques, the efficacy and complications of RP have also been improved [8]. According to several studies, RP represented better results of overall and cancer-specific survival compared with radiotherapy in patients with localized or high-risk prostate cancer [9, 10]. However, the overall efficacy of RP versus EBRT for localized prostate cancer hasn’t been systematically analyzed.

Therefore, we performed a meta-analysis on the efficacy of RP versus EBRT (3DCRT or IMRT) by comparing overall survival (OS), cancer-specific survival (CSS), and biochemical disease-free survival (BDFS) for patients with localized prostate cancer based on previous studies.

## RESULTS

### Search results

**Figure 1:**
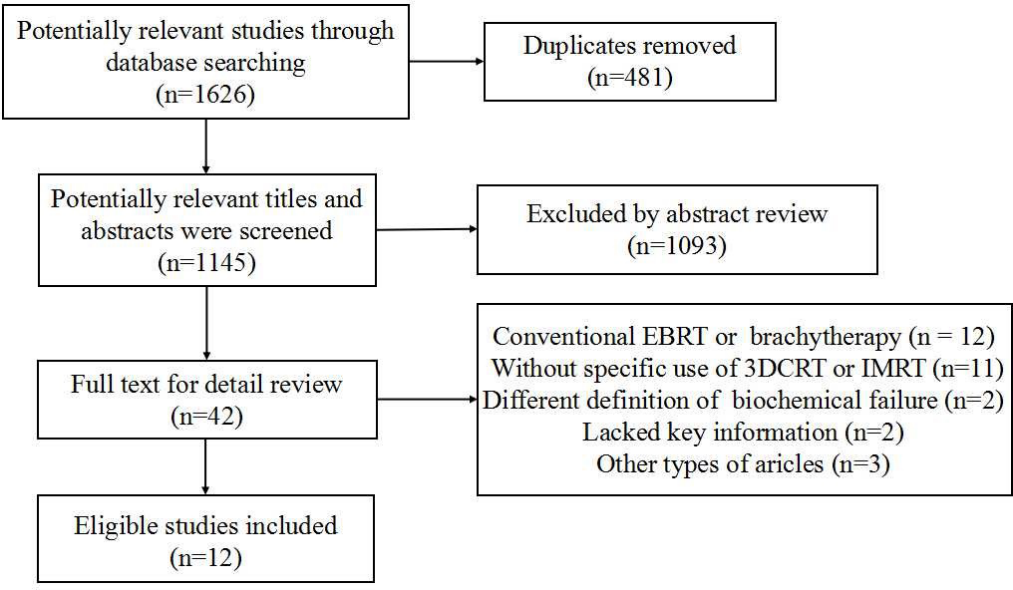
Study selection

As shown in Figure 1, a total of 1145 studies were searched from PubMed and EMBASE databases after duplicate articles were removed, and 42 articles were included for full-text review. Then 23 studies which contained patients treated with conventional EBRT or without specific results of 3DCRT or IMRT were excluded. And two articles were not eligible due to different definition of biochemical failure, which defined biochemical failure as PSA level of ≥ 0.4 ng/mL for postoperative patients. Finally, twelve studies with 17137 patients were identified.

### Characteristics and quality of studies

Among the 12 studies, patients of four cohorts [11–14] were treated with 3DCRT or RP, three studies [4, 15, 16] provided eligible data limited to IMRT, and other articles [17–21] contained both 3DCRT and IMRT. The radiation dose ranged from 70 Gy to 86.4 Gy, and patients treated with EBRT were mostly older than those who received RP. The adjuvant therapies were available in most included studies, but the use of androgen deprivation therapy (ADT) was considerably variable. There existed some studies with no or a few patients received ADT, while most of patients were treated with adjuvant therapy in other articles. Most studies did not provide the information of salvage therapies, and the use of salvage treatments was also different. In short, the main characteristics of included studies were represented on Table 1.

**Table 1:** The main characteristics of included studies

Author& Publication Year	Country (study interval)	Radiation Modality	Radiation dose (Gy)	Study Size (RP VS BRRT)	Age (RP VS EBRT)	Adjuvant Therapies (RP VS EBRT)	Salvage Therapies (RP VS EBRT)	Follow-up (RP VS EBRT)	Outcome
Aizer 2009	USA (1997–2005)	3DCRT+IMRT (32%), IMRT (68%)	75.6	204 VS 352	57.4 VS 68.4	ADT: 3% VS 80%	-	46 mo VS 60 mo	BDFS
Hamdy 2016	UK (1999–2009)	3DCRT	74	553 VS 545	62	-	RP: 0% VS 0.5% RT: 3 % VS 0% ADT: 1% VS 3%	10 yr	OS, CSS
Kibel 2012	USA (1995–2005)	3DCRT, IMRT	74	6485 VS 2264	61 VS 70	ADT: NA VS 34%	-	67 mo	OS, CSS
Kim 2014	Korea (2001–2011)	3DCRT (79%), IMRT (21%)	76	549 VS 189	66 VS 71	ADT: 27% VS 69%	-	48.8 mo VS 48.7 mo	OS, CSS, BDFS
Merino 2013	Chile (1999–2010)	IMRT	76	993 VS 207	63 VS 70	ADT: 0% VS 42%	RT: 5% VS NA	91.7 mo VS 76 mo	OS, CSS, BDFS
Nguyen 2008	USA (1965–2002)	3DCRT	70.2	659 VS 288	NA	ADT: 0% VS 0%	-	5.6 yr	CSS
Shinohara 2013	Japan (2003–2006)	IMRT	75 (65%); 70 (35%)	48 VS 23	67 VS 69	ADT: 0% VS 0%	-	73 mo VS 65 mo	BDFS
Taguchi 2015	Japan (2005–2012)	3DCRT (6%), IMRT (94%)	76	569 VS 322	66 VS 70	ADT: 24% VS 69.3%	RP: 0% VS 0% RT: 5% VS 0% ADT: 12% VS 7%	53 mo VS 45 mo	OS, CSS, BDFS
Takizawa 2009	Japan (1998–2004)	3DCRT	70–71	86 VS 76	64.9 VS 71.1	ADT: 83% VS 92%	-	5 yr	BDFS
Yamamoto 2013	Japan (1994–2005)	3DCRT	70	112 VS 119	67 VS 72	ADT: 2% VS 21%	RP: 0% VS 0% RT: 5% VS 0% ADT: 31% VS 27%	93 mo VS 85 mo	OS, CSS
Yamamoto 2016	Japan (2007–2013)	3DCRT (35%), IMRT (64%)	70–78	71 VS 43	70 VS 73	ADT: 0% VS 100%	-	59.1 mo VS 54.5 mo	BDFS
Zelefsky2 010	USA (1993–2002)	IMRT	81 (79%); 86.4 (21%)	1318 VS 1062	60 VS 69	ADT: 1% VS 56%	RP: 0% VS 0.3% RT: 4% VS 0% ADT: 4% VS 8%	5.1 yr VS 5.0 yr	CSS

Abbreviations: RP: radical prostatectomy; EBRT: external beam radiotherapy; 3DCRT: three-dimensional conformal radiotherapy; IMRT: intensity-modulated radiation therapy; ADT: androgen deprivation therapy; OS: overall survival; CSS: cancer-specific survival; BDFS: biochemical disease-free survival; NA: not available.

None of included studies was regarded as inferior quality with high risk of bias. However, some studies contained patients from different hospitals and lacked the information of the adequacy of follow-up, which may influence the quality of articles, and contribute to the risk of selection and attrition bias. We defined adequate duration of follow-up as the mean value more than 5 years, and most of included studies showed an adequate follow-up length. In brief, the quality assessment was showed in Table 4.

**Table 2:** The subgroup meta-analysis on survival outcome of HR following treatment with surgery or external beam radiotherapy

Factors	Overall survival (OS)			Cancer-specific survival (CSS)			Biochemical disease-free survival (BDFS)		
	Studies size	HR (95% CI, *p* value)	I^2^	Studies size	HR (95% CI, *p* value)	I^2^	Studies size	HR (95% CI, *p* value)	I^2^
**All studies**	**6**	**1.60 (1.44–1.79, *p* < 0.00001)**	**26%**	**8**	**1.73 (1.34–2.24, *p* < 0.0001)**	**0%**	**7**	**0.65 (0.51–0.82, *p* = 0.0004)**	**66%**
**Radiation modality**									
IMRT	1	1.75 (1.25–2.44, *P* = 0.001)	-	2	2.27 (1.23–4.20, *P* = 0.009)	0%	2	0.72 (0.55–0.94, *P* = 0.02)	0%
3DCRT	2	1.30 (0.96–1.75, *P* = 0.08)	75%	3	1.70 (1.11–2.62, *P* = 0.02)	0%	1	0.58 (0.33–1.02, *p* = 0.06)	-
**Risk stage**									
Low risk	3	1.76 (1.40–2.21, *p* < 0.00001)	0%	2	0.65 (0.07–6.10, *P* = 0.71)	73%	5	0.62 (0.33–1.15, *p* = 0.13)	59%
Intermediate risk	3	1.72 (1.09–2.69, *p* = 0.02)	52%	4	2.66 (0.73–9.64, *P* = 0.14)	73%	5	0.60 ( 0.47–0.77, *p* < 0.0001)	46%
High risk	3	1.79 (1.47–2.17, *p* < 0.00001)	0%	4	1.42 (1.13–1.77, *P* = 0.002)	0%	5	0.53 (0.44–0.64, *p* < 0.00001)	34%
**Median follow-up**									
< 5 year	2	1.72 (1.40–2.11, *p* < 0.00001)	0%	2	2.07 (0.81–5.34, *P* = 0.13)	47%	3	0.57 (0.46–0.72, *p* < 0.00001)	31%
5–7 year	1	1.60 (1.37–1.87, *p* < 0.00001)	-	3	1.83 (1.33–2.51, *P* = 0.0002)	21%	3	0.78 (0.39–1.55, *p* = 0.48)	83%
> 7 year	3	1.50 (1.02–2.20, *p* = 0.04)	65%	3	1.45 (0.88–2.37, *P* = 0.14)	0%	1	0.71 (0.54–0.94, *P* = 0.02)	-

Abbreviation: 3DCRT: three-dimensional conformal radiotherapy; IMRT: intensity-modulated radiation therapy.

**Table 3:** The subgroup meta-analysis on survival outcome of OR following treatment with surgery or external beam radiotherapy

OR	Factors	Overall survival (OS)			Cancer-specific survival (CSS)			Biochemical disease-free survival (BDFS)		
		Studies size	OR (95% CI, *p* value)	I^2^	Studies size	OR (95% CI, *p* value)	I^2^	Studies size	OR (95% CI, *p* value)	I^2^
**2-yr**	**All studies**	**5**	**1.56 (1.13–2.17, *p* = 0.008)**	**41%**	**6**	**1.27 (0.70–2.31, *P* = 0.42)**	**0%**	**6**	**0.21 (0.13–0.35, *p* < 0.00001)**	**65%**
	Low risk	2	6.04 (0.99–36.78, *p* = 0.05)	0%	0	All no death	-	5	0.31 (0.14–0.71, *p* = 0.006)	0%
	Intermediate risk	2	1.93 (0.43–8.64, *p* = 0.39)	0%	1	3.35 (0.30–37.41, *P* = 0.33)	-	5	0.25 (0.09–0.66, *p* = 0.005)	54%
	High risk	2	2.19 (0.67–7.16, *p* = 0.19)	0%	3	1.55 (0.54–4.44, *P* = 0.42)	0%	5	0.16 (0.07–0.36, *p* < 0.0001)	77%
**5-yr**	**All studies**	**5**	**3.18 (1.89–5.36, *p* < 0.0001)**	**77%**	**6**	**1.96 (1.42–2.72, *P* < 0.0001)**	**0%**	**7**	**0.42 (0.23–0.76, *p* = 0.004)**	**88%**
	Low risk	3	3.48 (1.63–7.44, *p* = 0.001)	38%	1	4.95 (0.30–80.82, *P* = 0.26)	-	5	0.35 (0.07–1.69, *p* = 0.19)	77%
	Intermediate risk	3	4.67 (2.51–8.71, *p* < 0.00001)	0%	2	4.52 (0.68–30.15, *p* = 0.12)	0%	5	0.46 (0.22–0.94, *p* = 0.03)	71%
	High risk	3	2.90 (1.73–4.87, *p* < 0.0001)	0%	3	1.55 (0.76–3.17, *p* = 0.23)	0%	5	0.26 (0.13–0.55, *p* = 0.0004)	83%
**10-yr**	**All studies**	**4**	**2.55 (1.63–3.99, *p* < 0.0001)**	**85%**	**6**	**2.44 (1.33–4.48, *p* = 0.004)**	**84%**	-	-	-
	Low risk	1	2.49 (0.84–7.42, *p* = 0.10)	-	1	0.80 (0.09–6.86, *p* = 0.84)	-	-	-	-
	Intermediate risk	1	2.35 (1.18–4.66, *p* = 0.01)	-	1	9.28 (2.04–42.28, *p* = 0.004)	-	-	-	-
	High risk	1	2.92 (1.60–5.35, *p* = 0.0005)	-	2	2.11 (0.71–6.30, *p* = 0.18)	70%	-	-	-

**Table 4:** Newcastle-Ottawa scale for quality assessment of included studies

Study	Selection				Comparability	Outcome			Overall
	Representativeness of exposed cohort	Selection of nonexposed	Ascertainment of exposure	Outcome not present at start		Assessment of outcome	Adequate follow-up length	Adequacy of follow-up	
Aizer 2009	✰	✰	✰	✰	✰✰	✰	✰		8
Hamdy 2016	✰	✰	✰	✰	✰✰	✰	✰	✰	9
Kibel 2012	✰	✰	✰	✰	✰✰	✰	✰		8
Kim 2014	✰	✰	✰	✰	✰✰	✰		✰	7
Merino 2013	✰	✰	✰	✰	✰✰		✰		7
Nguyen 2008	✰		✰	✰	✰✰		✰	✰	7
Shinohara 2013	✰	✰	✰	✰	✰✰		✰		7
Taguchi 2015	✰	✰	✰	✰	✰✰	✰			7
Takizawa 2009	✰	✰	✰	✰	✰✰		✰	✰	8
Yamamoto 2013	✰	✰	✰	✰	✰✰	✰	✰	✰	9
Yamamoto 2016	✰	✰	✰	✰	✰✰	✰			7
Zelefsky 2010	✰		✰	✰	✰✰	✰	✰	✰	8

### Overall survival of RP versus EBRT

**Figure 2:**
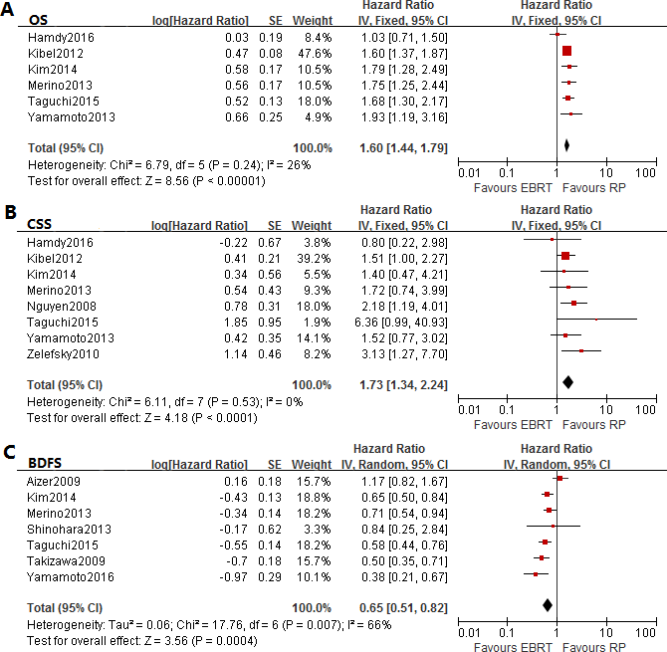
Forest plots of hazard ratio (HR) for overall survival (OS) **(A)**, cancer-specific survival (CSS) **(B)**, and biochemical disease-free survival (BDFS) **(C)**.

Six studies provided the data of OS between postoperative patients and post-radiotherapy patients , and the combined HR and 95% CI was 1.60 (1.44–1.79, *p* < 0.00001) without significant heterogeneity (I^2^ = 26%) (Figure 2A; Table 2). In terms of low-risk, intermediate-risk and high-risk patients, the results of subgroup analyses represented similar HR of 1.76 (95% CI: 1.40–2.21, *p* < 0.00001; I^2^ = 0%), 1.72 (95% CI: 1.09–2.69, *p* = 0.02; I^2^ = 52%) and 1.79 (95% CI: 1.47–2.17, *p* < 0.00001; I^2^ = 0%), respectively. Only a study compared the OS between IMRT and RP (HR = 1.75, 95% CI: 1.25–2.44, *P* = 0.001), and there was no significant differences of 3DCRT versus RP (*P* = 0.08, I^2^ = 75%). Meanwhile, the results were still significant according to analyses of median follow-up (Table 2).

**Figure 3:**
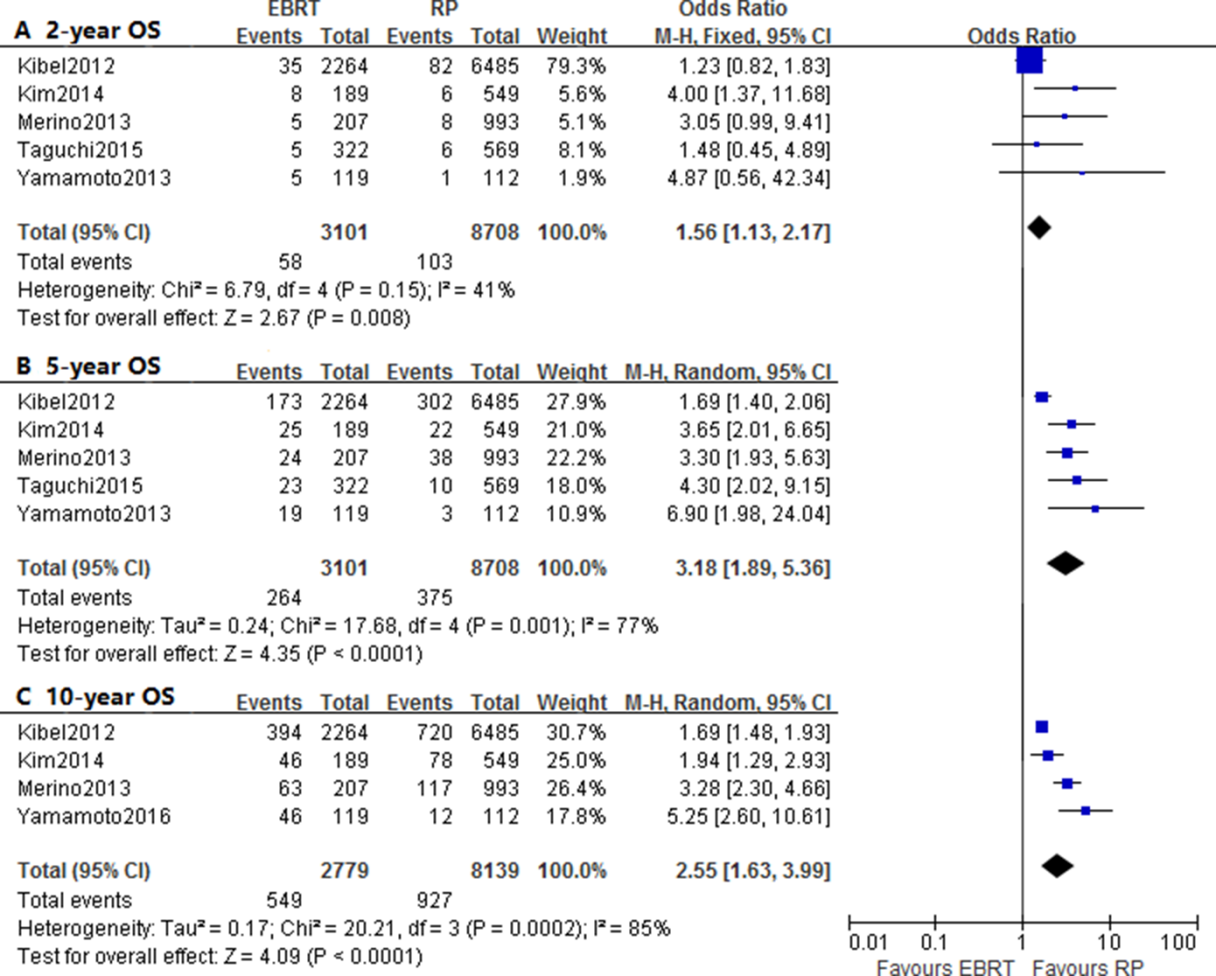
Forest plots of odd ratio (OR) for 2-year **(A)**, 5-year **(B)** and 10-year **(C)** overall survival (OS).

The pooled OR and 95% CI on 2-year, 5-year and 10-year OS were 1.56 (1.13–2.17, *p* = 0.008), 3.18 (1.89–5.36, *p* < 0.0001) and 2.55 (1.63–3.99, *p* < 0.0001), respectively. There was significant heterogeneity with respect to 5-year OS (I^2^ = 77%) and 10-year OS (I^2^ = 85%) (Figure 3; Table 3). As for the subgroup analyses based on risk stage, there were not enough studied provided with 10-year survival outcome, and only 5-year OS results of of risk stratification was significant.

### Cancer-specific survival of RP versus EBRT

Seven studies were involved in the assessment, which indicated the patients may benefit from RP with a relatively lower risk of cancer-specific mortality (HR = 1.73, 95% CI: 1.34–2.24, *p* < 0.0001; I^2^ = 0%) (Figure 2B; Table 2). However, the HR and 95% CI were 0.65 (0.07–6.10, *P* = 0.71; I^2^ = 73%), 2.66 (0.73–9.64, *P* = 0.14; I^2^ = 73%) and 1.42 (1.13–1.77, *P* = 0.002; I^2^ = 0%) according to low-risk, intermediate-risk and high-risk stages, and only the high-risk patients represented a significant difference. In addition, only group with median follow-up of 5–7 years reflected significant difference (*p* = 0.0002; I^2^ = 21%). We observed significant differences between post- IMRT patients and post-RP patients (*p* = 0.02; I^2^ = 0%), and patients treated with 3DCRT versus RP (*P* = 0.009; I^2^ = 0%) (Table 2).

**Figure 4:**
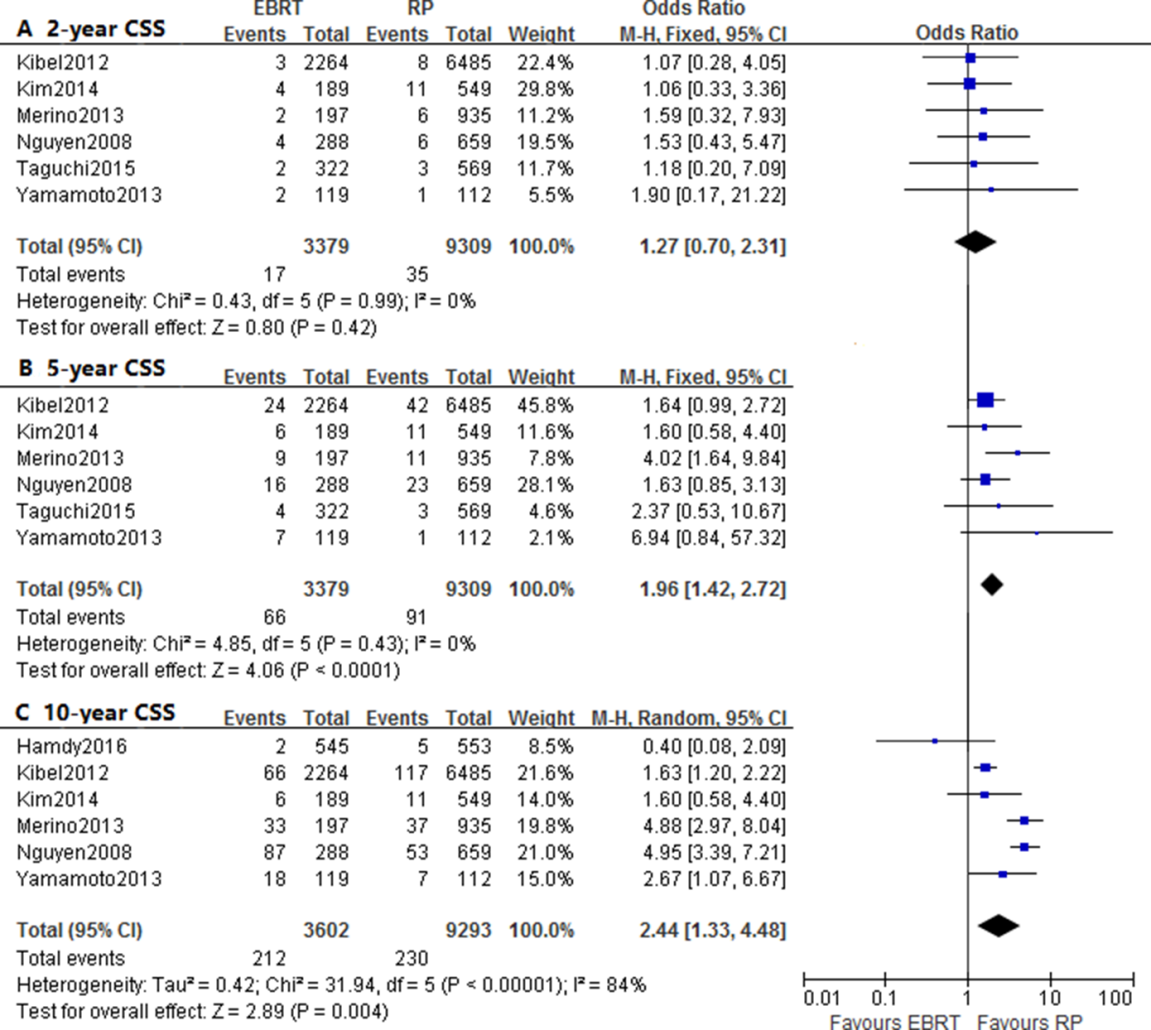
Forest plots of odd ratio (OR) for 2-year **(A)**, 5-year **(B)** and 10-year **(C)** cancer-specific survival (CSS).

The analyses on 2-year, 5-year and 10-year CSS reported the OR and 95% CI of 1.27 (0.70–2.31, *P* = 0.42), 1.96 (1.42–2.72, *P* < 0.0001) and 2.44 (1.33–4.48, *p* = 0.004), respectively. There was significant heterogeneity observed when assessed the OR of 10-year CSS (I^2^ = 84%) (Figure 4; Table 3). Two studies provided the CSS of risk stratification, and one studies only reflected 2-year and 5-year survival results. However, there was no death of low or intermediate-risk groups based on two studies, thus significant differences were not found, which may also due to insufficient eligible data.

### Biochemical disease-free survival of RP versus EBRT

In this comparison, seven studies were aggregated to analyze the influence of treatment modality on BDFS, and the pooled HR was 0.65 (95% CI: 0.51–0.82, *p* = 0.0004; I^2^ = 66%) (Figure 2C; Table 2) , which reflected additional biochemical survival benefiting from EBRT than RP. Moreover, the subgroup comparison of radiation modality also suggested a better BDFS in patients treated with IMRT (*P* = 0.02; I^2^ = 0%). And the statistical results of intermediate-to high-risk groups showed similar direction of effect with HR of 0.60 (0.47–0.77, *p* < 0.0001; I^2^ = 46%) and 0.53 (0.44–0.64, *p* < 0.00001; I^2^ = 34%), but low-risk group failed to show significant differences (*p* = 0.13; I^2^ = 59%) In addition, only subgroup with median follow-up less than 5 years reflected significant difference (*p* < 0.00001; I^2^= 31%) (Table 2).

**Figure 5:**
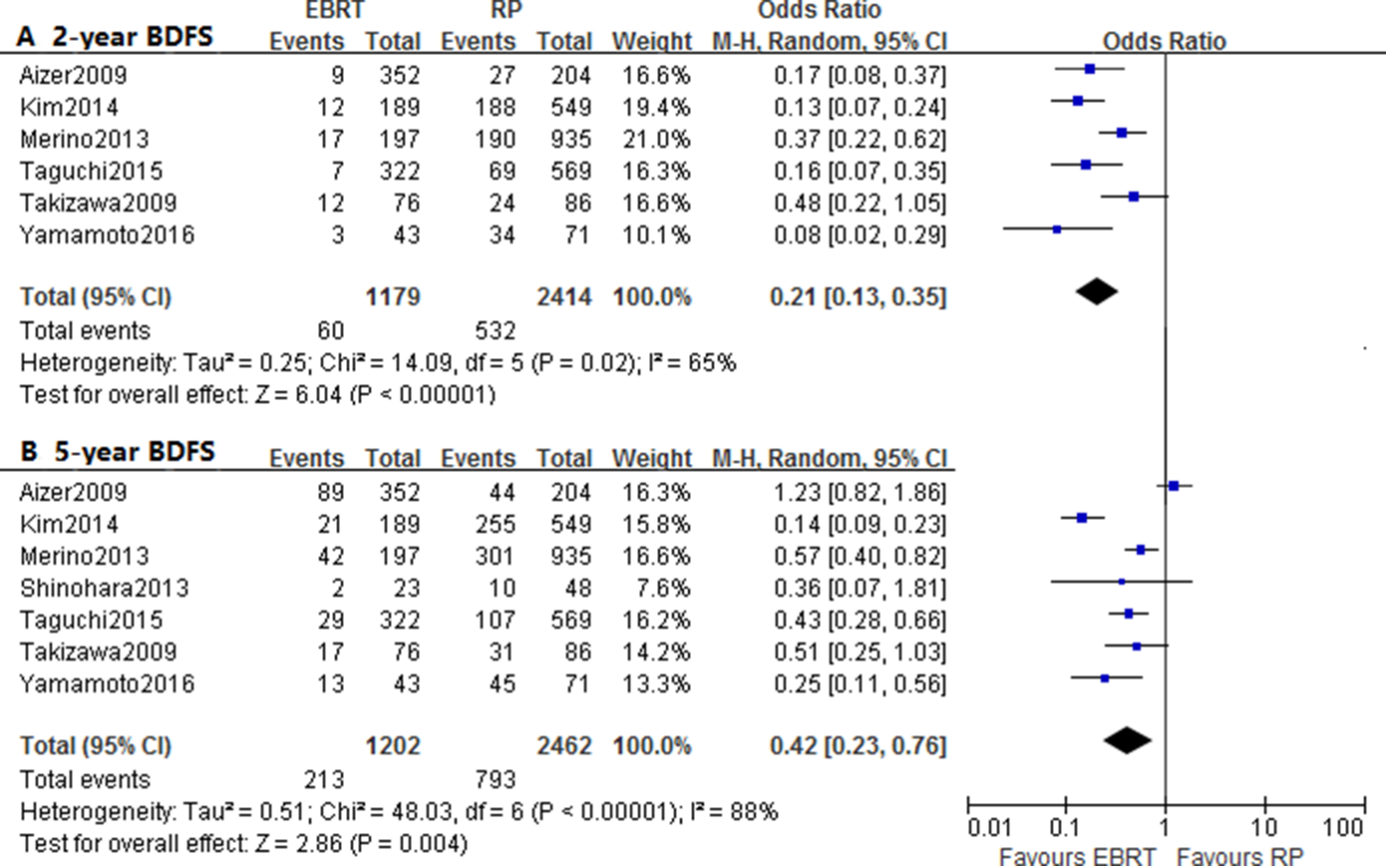
Forest plots of odd ratio (OR) for 2-year **(A)** and 5-year **(B)** biochemical disease-free survival (BDFS).

The included studies could only provide with data of 2-year and 5-year BDFS, and the pooled OR and 95% CI were 0.21 (0.13–0.35, *p* < 0.00001; I^2^ = 65% ) and 0.42 (0.23–0.76, *p* = 0.004; I^2^ = 88%) (Figure 5; Table 3). The results of subgroup analyses showed significant differences, except for 5-year BDFS of low-risk patients.

### Publication bias

We performed assessment of publication bias by Begg’s test, and suggested no evident publication bias in OS (*P* = 0.339), CSS (*P* = 0.368) and BDFS (*P* = 0.613). In addition, there were no significant bias identified in the 2-year, 5-year and 10-year OS, CSS and BDFS.

## DISCUSSION

For patients with localized prostate cancer, there was no final consensus of the most optimal treatments. According to previous randomized cohorts, both RP and EBRT were effective in decreasing cause-specific mortality or cancer progression with comparison to watchful waiting [11, 22]. And the established clinical guidelines advised patients which desiring non-conservative intervention to choose treatment option themselves [23]. Thus we performed this meta-analysis to help reveal the effectiveness of EBRT (3DCRT and IMRT) with surgery as the control.

The 2-year, 5-year and 10-year OR of OS, CSS and BDFS were used to estimate the differences of short-term, mid-term and long-term survival between RP and EBRT. And considering existed controversy over the efficacy, we also performed analysis of HR to compare the risk between treatment modality. Firstly, the ORs supported that patients benefited from RP in terms of 2-year, 5-year and 10-year overall survival. According to HR, a reduced risk of overall mortality was also identified for post-RP patients, which was in accordance with subgroup analyses of risk stage. The post-EBRT patients with low-to high-risk prostate cancer all showed an increased risk of overall mortality than post-RP patients.

Meanwhile, there were significant differences in the OR of 5-year and 10-year CSS, which indicated that the RP group patients had better results of the mid-term and long-term survival. Many prostate cancers were indolent tumor with low cancer-sepcific mortality, and patients showed relatively longer survival length, which may explained the insignificant differences of 2-year CSS. On the other hand, RP was associated with lower risk of cancer-specific mortality based on HR, which was consistent with the conclusion of a previous meta-analysis containing patients treated with all kinds of radiotherapy or surgery [9]. However, with risk stratification, only high-risk group revealed significant difference, and the efficacy was not considerably different in low-to intermediate-risk patients. The combined HR of studies with median follow-up length more than 7 year also failed to show statistically significance. As shown in table 1, the EBRT group patients were considerably older than RP group patients, and older patients had higher risk of comorbidities, which may contribute to the worse OS and CSS. In addition, the different use of salvage therapies after biochemical failure might explain the survival differences, because post-PR patients had more treatment options such as salvage radiotherapy or ADT, but fewer post-EBRT patients received salvage surgery. Therefore, considering the relatively small amount of included studies in subgroup analyses, more high-quality prospective studies were requisite to assess the efficacy of RP versus EBRT based on varied conditions.

The biochemical survival was significantly different based on HR. The OR of 2-year and 5-year BDFS also showed a better result among patients received EBRT, which was consistent with subgroup analyses of risk stratification. The different nadir levels were used in defining the biochemical failure, the post-radiotherapy patients were regarded as biochemical failure with higher PSA level, thus the result of BDFS might not reflect the mortality [20]. And the higher rate of biochemical failure for postoperative patients might contribute to the higher use of salvage therapies.

Treatment-related toxicity should also be assessed. Among the included articles, two studies [15, 21] reported the adverse events of EBRT, which mostly consisted of diarrhea, anal pain and urinary frequency. Another studies [13] compared the health-related quality of life and functional outcomes of patients treated with RP or EBRT. Accordingly, after the RP therapy, the low-to intermediate-risk patients reported decreased urinary function, and high-risk patients were related with worse sexual function. An included study also indicated that IMRT had less negative effect on sexual and urinary function [15]. As for patients with high-risk prostate cancer, RP was recommended as the first option refers to AUA guideline, and EAU also advised to receive RP followed by ADT [23, 24]. On the contrary, the NCCN guideline suggested EBRT with adjuvant ADT as optimal treatment [25]. According to our analytic result, RP might be a more appropriate option for high-risk patients, since RP was associated with reduced risk of overall and cancer-specific mortality as well as better 5-year and 10-year OS and CSS in general. But in terms of patients with low-to intermediate risk prostate cancer, EBRT might be considered as viable alternative to RP. Recently, the randomized study by Hamdy et al. [11] concluded that there were no significant difference of CSS between RP, EBRT and active monitoring, and the mortality from prostate cancer remained low within 10 years of follow-up. However, RP and EBRT contributed to the decreased incidences of cancer progression and metastasis. As the only randomized clinical trial of RP and EBRT on prostate cancer, this study may also help the clinical decision making. And it is important to enroll more patients on large-scale randomized trials to comprehensively compare outcomes of different treatments. In general, it suggested making the treatment decision based on several aspects including patient functional condition, cost effectiveness, complication and risk of surgery, treatment-related side effects and personal preference.

Statistically significant heterogeneity was identified in the analytic results of BDFS, OS and CSS, which may due to the uncertainty of survival estimation including administration death records and determination by physician. And the different study design such as sample size, use of adjuvant and salvage therapies, patients’ characteristics (age, tumor stage, risk classification and complications) may also result in the heterogeneity between included studies.

There were several limitations in this meta-analysis. First of all, the differences of included studies were inevitable, which may be due to different patients’ characteristics. For instance, older patients were more frequently referred to radiotherapy, thus patients who received RP were significantly younger than those treated with EBRT, as shown in Table 1. And post-PR patients had more treatment options than post-EBRT patients, such as salvage radiotherapy or ADT. The potential comorbidities of patients could also influence the survival results. Secondly, ADT has been a first-line treatment for more than half a century, which might have function of eliminating residual tumor cells in the primary and metastatic lesions [26]. However, the use of ADT as adjuvant or salvage therapy in included studies was considerably variable. Then this meta-analysis was limited in English articles, and most included studies were retrospective cohort studies. In addition, for advanced EBRT such as IMRT, comparison of EBRT versus RP should be updated, but the studies of IMRT versus surgery were not adequate. Therefore, high-quality and long-term prospective randomized cohorts based on large amount of patients were need for comparison and contributed to the clinical decision.

## MATERIALS AND METHODS

### Search strategy

Articles about the comparison of efficacy between treatments were searched from PubMed and EMBASE databases until December, 2016 using relevant terms and medical keywords as follow: “prostate cancer”; “radiotherapy” or “external beam radiotherapy” or “EBRT”; “radical prostatectomy” or “surgery” and “survival” or “mortality”.

### Selection criteria

Studies were considered eligible refers to the following criteria: (1) the included patients were localized prostate cancer, (2) the articles researched the efficacy between radical prostatectomy and external beam radiotherapy (3DCRT or/and IMRT), (3) the data of survival outcomes (OS, CSS or BDFS) was provided, (4) the biochemical failure was defined as the PSA level which greater than or equal to 0.2 ng/mL for post- RP patients and 2 ng/mL for post-EBRT patients [27].

Studies were excluded according to the following exclusion criteria: (1) the studies included patients treated with conventional EBRT or brachytherapy, or didn’t provide the specific instructions about the use of 3DCRT or IMRT, (2) the studies lacked key information of survival results, (3) the other types of articles contained reviews, letters and case reports, (4) the studies were non-English articles.

### Data extraction

Main information of eligible studies were extracted according to pre-defined tables, which included: (1) the basic information: first author, publication year, country and study interval; (2) the characteristics of cohorts: patients’ size and age, radiation modality and dose, duration of follow-up, use of adjuvant and salvage therapies , and (3) survival outcome.

### Quality assessment

We used Newcastle-Ottawa Scale (NOS) to assess the quality of non-comparative studies [28]. A maximum of 1 star can be allotted in seven aspects of selection and outcome, and the comparability of groups accounted for 2 stars at most, thus the quality scores ranged from 0 to 9. Accordingly, the scores which great than or equal to 7 represented a high quality and low risk of bias, and the studies with scores less than 4 were considered as inferior quality with high risk of bias.

### Statistical analysis

Both hazard ratio (HR) and odd ratios (OR) were used to analyze the survival outcomes. The HR was calculated by the *p*-value of log-rank test and the events of Kaplan-Meier survival curves on Engauge Digitizer 4.1 when the original studies did not provide with direct data [29]. In addition, a pooled HR or OR > 1 without 95% CI overlapping 1 was considered as statistically significant if *p* < 0.05. In terms of *X*^2^ test, the non-significant heterogeneity was defined as *p* ≥ 0.10 or I^2^ ≤ 50%, and a fixed effect model was selected [30]. In contrast, a random effect model was used if the heterogeneity was significant (*p* < 0.10 or I^2^ > 50%). The meta-analysis of HR and OR was also performed by subgroup of radiation modality, risk stage or/and median duration of follow-up. All the above analyses were performed by Review Manager 5.2 software. We used Begg’s test [31] to estimate the publication bias by means of STATA 11.0 and the evidence of significant publication bias was not existed if *p* > 0.05.

## CONCLUSIONS

In summary, RP was related with decreased risk of overall and cancer-specific mortality as well as better 5-year and 10-year OS and CSS in general. There were no significant differences of CSS for low- to intermediate-risk patients, which suggested EBRT might as a promising alternative option with better post-treatment quality of life and functional outcomes. Considering the inconsistency of subgroup analyses, large-scale prospective studies with risk stratification and adequate duration of follow-up were needed to attain a comprehensive comparison between RP and EBRT for localized prostate cancer.
